# Family-Wide Survey of *miR169s* and *NF-YAs* and Their Expression Profiles Response to Abiotic Stress in Maize Roots

**DOI:** 10.1371/journal.pone.0091369

**Published:** 2014-03-14

**Authors:** Mingda Luan, Miaoyun Xu, Yunming Lu, Qiuxue Zhang, Lan Zhang, Chunyi Zhang, Yunliu Fan, Zhihong Lang, Lei Wang

**Affiliations:** 1 School of Life Science and Engineering, Southwest University of Science and Technology, Mianyang, China; 2 Biotechnology Research Institute/The National Key Facility for Crop Gene Resources and Genetic Improvement, Chinese Academy of Agricultural Sciences, Beijing, China; 3 Shenzhen Nongke Group CO., LTD, Shenzhen, China; Universidade Federal do Rio Grande do Sul, Brazil

## Abstract

Previous studies have identified miR169/NF-YA modules are important regulators of plant development and stress responses. Currently, reported genome sequence data offers an opportunity for global characterization of *miR169* and *NF-YA* genes, which may provide insights into the molecular mechanisms of the *miR169/NF-YA* modules in maize. In our study, fourteen NF-YA transcription factors with conserved domains were identified based on maize genome loci. The *miR169* gene family has 18 members that generate 10 mature products, and 8 of these mature miR169 members could target 7 of 14 *ZmNF-YA* genes in maize. The seven ZmNF-YA proteins were localized to the nucleus while lacked transcriptional activity. We investigated the expression patterns of the *zma-miR169* members and their targeted *ZmNF-YA* genes in maize roots treated by drought stress (polyethylene glycol, PEG), hormone stress (abscisic acid, ABA), and salt stress (NaCl). The *zma-miR169* family members were downregulated in short term (0∼48 h) and generally upregulated over the long term (15 days) in response to the three abiotic stress conditions. Most of the targeted *ZmNF-YA* genes exhibited a reverse correlation with *zma-miR169* gene expression over both the short term and long term. Maize root elongation was promoted by PEG and ABA but repressed by NaCl over the long term. Apparently, *ZmNF-YA14* expression perfectly matched the *zma-miR169* expression and corresponded to root growth reversely.

## Introduction

In recent years, the miRNA/target module has been shown to be involved in regulatory cascades in plant development and biotic and abiotic stress responses [1]–[3] in which miRNAs mediate transient gene silencing [4], [5]. The miRNA/target module cascade plays an important role on responding to stress besides coordinating normal growth and development [6]. In *Arabidopsis*, for instance, miRNA expression is either up- or downregulated depending on the particular stress condition with their targets being inhibitors of stress responses or components of stress-inhibited processes [4], [5].

Understanding small RNA-guided stress regulatory networks can provide new insights for the genetic improvement of stress tolerance in plants. Many studies have revealed complexity and overlap in plant responses to different stresses. Understanding this complexity and overlap would lead to new ways to enhance crop tolerance to diseases and environmental stress. Manipulation of miRNA-guided gene regulation can help in the engineering of stress-resistant plants [7]. While some miRNA families have functions that are conserved in many plant species, other stress-responsive miRNA families may exhibit distinct expression profiles in different plant species or even in related genotypes of the same species that have distinct stress sensitivities [6], [8]. Further in-depth analysis is required to clarify these apparent contradictions in miRNA-expression profiles during plant stress responses [6].

The miR169 family is the largest and most conserved miRNA family in plants and has been shown to be involved in plant responses to abiotic stress [3], [9]–[11]. Eighteen *miR169* family members are present in maize (zma-miR169). How the *zma-miR169* family members respond to drought or salt stress or exogenous abscisic acid (ABA) treatment has not been reported.

Computational prediction and experimental analyses suggest that *miR169* targets members of the *NF-YA* gene family [12]–[14]. *NF-Y* genes encode a CCAAT-binding transcription factor, which participates in transcriptional regulation of a large number of genes. Genes encoding NF-Y transcription factors are found in all eukaryotes. The NF-Y family includes at least three subunits, NF-YA, NF-YB, and NF-YC, all of which are required for CCAAT binding and downstream gene transcription. During transcriptional activation, NF-YA, NF-YB, and NF-YC form a heterotrimer [15]. Each NF-Y subunit is encoded by a single gene in yeast and animals, but in plants, each NF-Y subunit is encoded by a multigene family. At least 10 *NF-YA* genes, 12 *NF-YB* genes, and 8 *NF-YC* genes are present in rice [16]. In maize, 36 potential *NF-YA* genes, 28 potential *NF-YB* genes, and 25 potential *NF-YC* genes are in the Plant Transcription Factor Database (http://planttfdb.cbi.pku.edu.cn/). NF-YB and NF-YC form a dimer in the cytoplasm and then translocate to the nucleus to join with NF-YA to form a trimer, which binds the 25-bp CCAAT-box as a regulator of downstream genes. In this process, the complete trimer makes the promoter region of the chromatin accessible to other regulatory factors, and NF-YA acts as a CCAAT motif seeker that can insert into the minor groove of the DNA [17]. No NF-YA/B/C complex has been associated with the regulation of the expression of a particular gene or process in plants. Single plant *NF-YA* genes are known to have functions in nodule development [18], N deficiency [19], and ABA response [20]. Ath-miR169a/AtNF-YA5 module was involved in drought tolerance [10]. In addition, ath-miR169d/AtNF-YA2 module was shown to be involved in stress-induced early flowering in *Arabidopsis* [21]. To date, no *NF-YA* genes or miR169/NF-YA modules in maize have been found to regulate responses to stress caused by drought, salt, or ABA.

In our study, we explored a new classification for the *ZmNF-YA* family members based on gene loci and conserved domains. We predicted and confirmed that the mRNAs of seven *ZmNF-YA* genes were cleaved by *zma-miR169s*. Additionally, the subcellular localization and transcriptional activation activities of *zma-miR169* targeted ZmNF-YAs were investigated. We defined the expression profiles of mature *zma-miR169* family members and their target *ZmNF-YA* genes in maize roots in response to three abiotic stress conditions.

## Results

### Characterization of the maize *miR169* family

**Figure 1:**
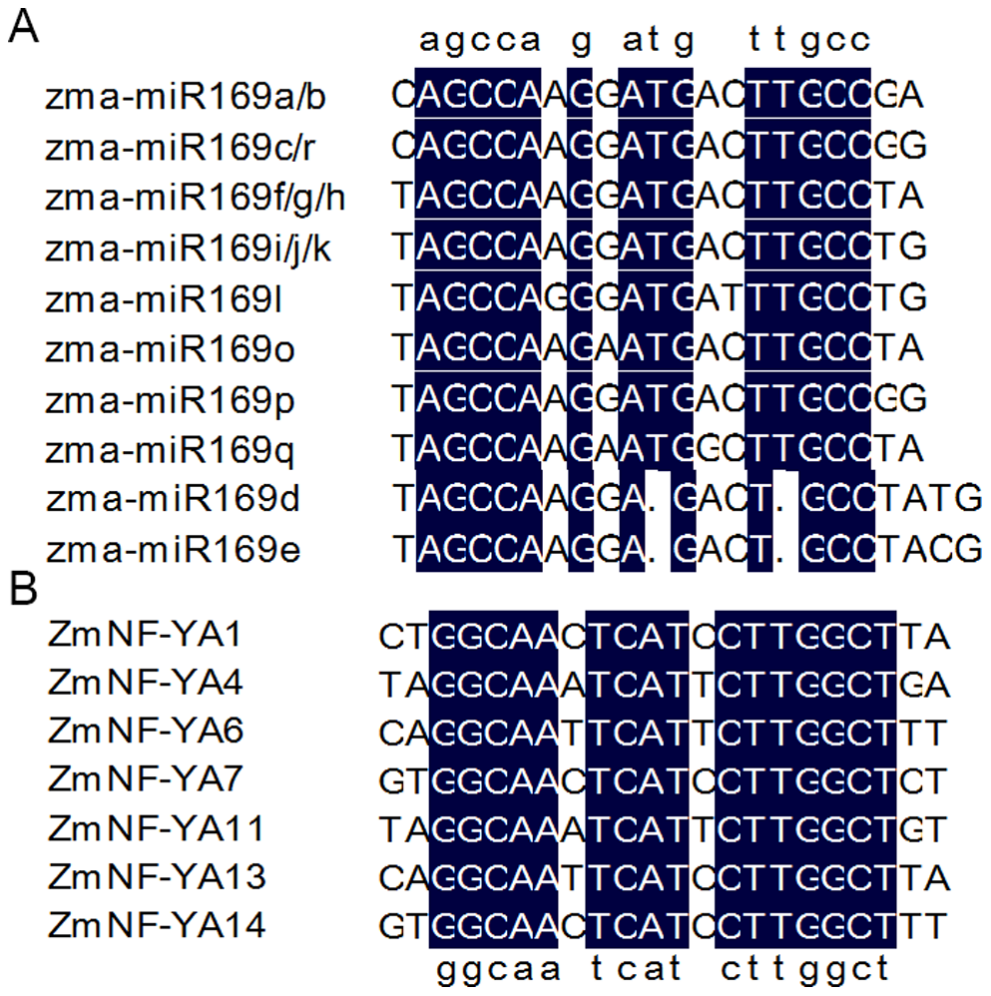
Nuclear acid sequence alignments of mature zma-miR169 (A) and zma-miR169 target sites in ZmNF-YA family members (B). *Zma-miR169d* and *zma-miR169e* are deleted at sequence 11.

According to miRBase version 20 (http://www.mirbase.org/) and published data, the miR169 family is large and conserved, and found in 35 plant species, including monocots and dicots as well as some ancient gymnosperms (Table S1). This ubiquity highlights its critical regulatory roles in plants. We found that 18 *miR169* family members were located on eight chromosomes in maize (Table S2) and produced 10 mature products with high similarity (Figure 1). Outside of the mature miRNA regions, *zma-miR169* family precursors differed widely in size and consensus sequences (Figure S1). Three conserved motifs exist: “AGCCA”, “ATG” and “TTGCC” in the mature *zma-miR169* sequences except in *zma-miR169d* and *zma-miR169e*. The “ATG” motif located at nucleotides 10 and 11, which is significant to miRNA cleavage [22]. Exactly equivalent conserved motifs were present in *miR169* target sites located in 3′ UTR region of *ZmNF-YA* (Figure 1B, addressed in following section). For *zma-miR169d* and *zma-miR169e*, considering the deletion T at position 11 (Figure 1), we concluded that *zma-miR169d* and *zma-miR169e* may function as translational inhibition factors or are targeted to other genes.

### Characterization of the NF-YA family in maize

**Figure 2:**
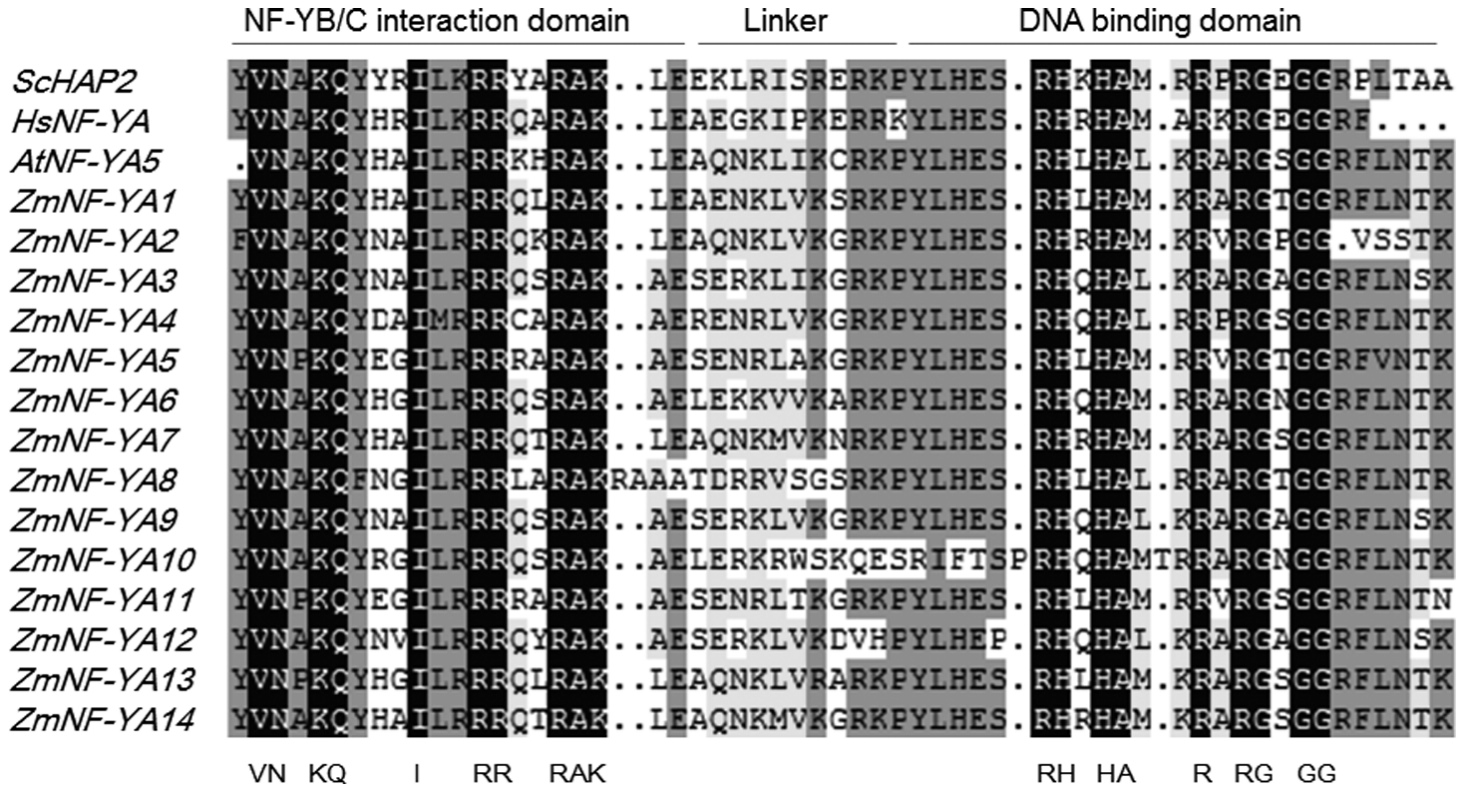
Amino acid alignment of NF-YA core domains from Sc, Saccharomyces cerevisiae; At, Arabidopsis; Hs, Homo sapiens; Zm, Zea mays.

NF-YA is one subunit of the NF-Y complex, a universal transcriptional factor that binds the CCAAT box in eukaryotes. Thirty-six potential NF-YA family members are present in the plantTFDB database (Figure S2). Based on gene loci and their chromosome positions [23], and conserved functional domains of the NF-YA family, we identified and renamed 14 *ZmNF-YA* loci (Table 1). The NF-YA core domain is less than 60 amino acids long and is sufficient for DNA binding when complexed with NF-YB/NF-YC [24]. The NF-YA core domain consists of three motifs: an N-terminal motif responsible for NF-YB/NF-YC binding, a C-terminal motif implicated in specific recognition of the CCAAT element, and a middle linker region (Figure 2). To evaluate conservation among different species, highly conserved domains (HCDs) of NF-YAs from *Saccharomyces cerevisiae*, *Homo sapiens*, *Arabidopsis thaliana*, and *Zea mays* were aligned (Figure 2). The N-terminal and C-terminal motifs were highly conserved in eukaryotes.

**Table 1:** Annotation of the NF-YA gene family members in maize.

NF-YA Name	Locus Name	Chromosome Position
*ZmNF-YA1 ZmNF-YA2 ZmNF-YA3 ZmNF-YA4 ZmNF-YA5 ZmNF-YA6 ZmNF-YA7 ZmNF-YA8 ZmNF-YA9 ZmNF-YA10 ZmNF-YA11 ZmNF-YA12 ZmNF-YA13 ZmNF-YA14*	GRMZM2G000686 GRMZM2G037630 GRMZM2G361842 GRMZM5G853836 GRMZM5G829103 GRMZM2G091964 GRMZM2G040349 GRMZM2G096016 GRMZM2G104396 GRMZM2G026157 GRMZM2G165488 GRMZM2G582893 GRMZM5G857944 GRMZM2G038303	Chromosome 1: 15,805,373–15,808,637 Chromosome 1: 71,589,264–71,595,809 Chromosome 1: 173,335,601–173,340,617 Chromosome 1: 174,875,434–174,876,987 Chromosome 1: 250,377,935–250,382,233 Chromosome 1: 263,505,895–263,513,358 Chromosome 2: 211,453,451–211,457,855 Chromosome 2: 235,103,649–235,106,245 Chromosome 3: 123,431,302–123,438,343 Chromosome 5: 12,557,664–12,558,570 Chromosome 5: 16,480,475–16,484,858 Chromosome 5: 22,308,191–22,311,425 Chromosome 5: 211,715,089–211,720,032 Chromosome 7: 165,030,959–165,035,270

Each gene was named with a two-letter species indicator corresponding to *Zea mays* (Zm), followed by the family designation (NF-YA) and a number based on chromosomal position.

**Figure 3:**
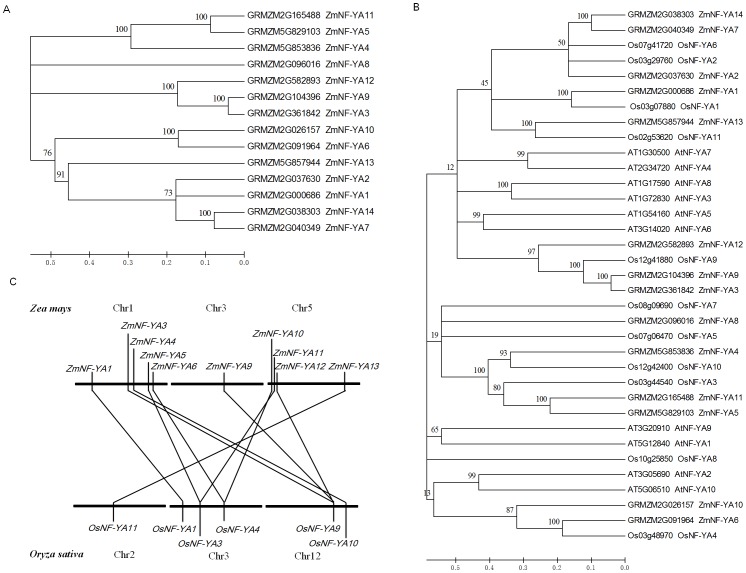
Phylogenetic analyses of NF-YA proteins. (A) Phylogenetic analysis of ZmNF-YA family members. (B) Phylogenetic analysis of NF-YA proteins from maize, rice, and Arabidopsis. (C) Syntenic relationships of NF-YA genes between maize and rice. Each line represents an orthologous gene. The loci of *NF-YA* genes involved are shown in Table S3. Phylogenetic trees were constructed by neighbor joining with complete deletions as implemented by Molecular Evolutionary Genetics Analysis software, version 5.0 (MEGA5) [33]. Reliability values at each branch represent bootstrap samples (1000 replicates).

To detect the intraspecific and interspecific evolutionary relationships of the NF-YA family members within maize and among maize, *Arabidopsis*, and rice, unrooted phylogenetic trees were constructed based on full-length protein sequences (Figure 3A, B). Apart from ZmNF-YA8, the maize NF-YA proteins fell into four obvious clades: ZmNF-YA4, 5, and 11 (clade I), ZmNF-YA3, 9, and 12 (clade II), ZmNF-YA6 and10 (clade III), and ZmNF-YA1, 2, 7, 13, and 14 (clade IV). Despite some differences outside the HCD sequences, members of clade I showed 87% sequence similarity and members of clades II and IV shared 93.5% and 53.3% sequence similarity, respectively (Figure S3). We concluded that the *ZmNF-YA* gene family has undergone a history of expansion due to duplication events and that different members may have evolved at different times and rates. Further analysis revealed that ZmNF-YA proteins had higher homology to NF-YA proteins in rice than in *Arabidopsis* (Figure 3B). Because maize and rice are monocots, we were interested in the evolutionary relationship of the *NF-YA* genes in maize and rice; thus, an interspecific comparison was performed (Figure 3C). Ten orthologous relationships covering three chromosomes were identified. The following chromosome-to-chromosome evolution relationships were established: *ZmNF-YA1*—*OsNF-YA1*, *ZmNF-YA3/ZmNF-YA9/ZmNF-YA12*—*OsNF-YA9*, *ZmNF-YA4*—*OsNF-YA10, ZmNF-YA5/ZmNF-YA11*—*OsNF-YA3*, *ZmNF-YA6/ZmNF-YA10*—*OsNF-YA4*, and *ZmNF-YA13*—*OsNF-YA11* (Figure 3C).

### Potential targets of the zma-miR169 family in maize

**Figure 4:**
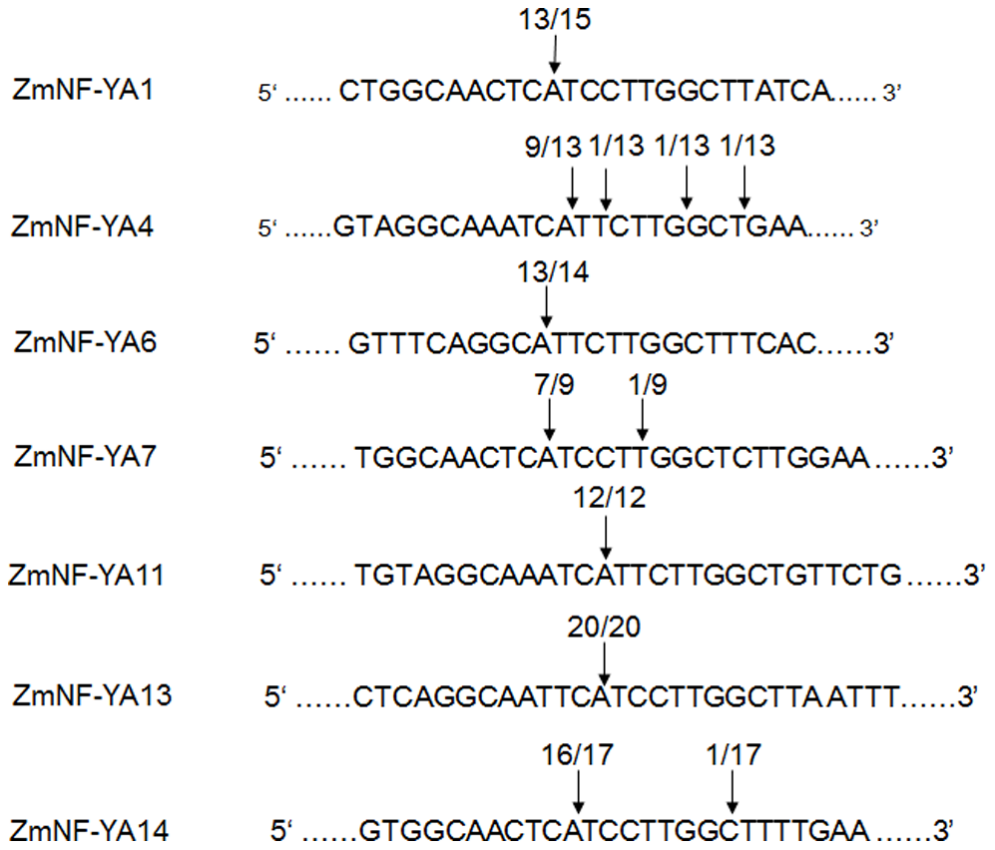
Mapping of Zm-NFYA cleavage sites generated by zma-miR169s. Cleavage sites are indicated by arrows. 5′ termini of mRNA fragments isolated from maize were determined from cloned 5′RACE products (Figure S4). The frequencies of cleavage site usage are indicated by fractional numbers.

Cleavage of *NF-YA* mRNAs is directed by *miR169* in *Arabidopsis thaliana*, *Oryza sativa*, and *Glycine max* [12]–[14]. *ZmNF-YA1*, *ZmNF-YA4*, *ZmNF-YA6*, *ZmNF-YA7*, *ZmNF-YA11*, *ZmNF-YA13*, and *ZmNF-YA14* mRNAs were predicted to be targets of *zma-miR169s* based on analysis using psRNA Target online software. The target sites were located in the 3′UTR regions of *ZmNF-YA* mRNAs and were conserved among different members (Table 2 and Figure 1B). We performed RLM-5′RACE to verify the predicted targets and cleavage sites. The mRNAs of all 7 *ZmNF-YAs* were cleaved at the predicted cleavage sites, demonstrating that *zma-miR169s* (except *zma-miR169d* and *zma-miR169e*) guided the cleavage of predicted targets (Figure 4 and Figure S4) [22].

**Table 2:** Predicted targets of mature zma-miR169s in maize.

miRNA	Predicted targets
*zma-miR169a/b*	*ZmNF-YA1; ZmNF-YA4; ZmNF-YA7; ZmNF-YA11; ZmNF-YA13; ZmNF-YA14;*
*zma-miR169c/r*	*ZmNF-YA1; ZmNF-YA4; ZmNF-YA7; ZmNF-YA11; ZmNF-YA13; ZmNF-YA14;*
*zma-miR169f/g/h*	*ZmNF-YA1; ZmNF-YA4; ZmNF-YA6; ZmNF-YA7; ZmNF-YA11; ZmNF-YA13; ZmNF-YA14;*
*zma-miR169i/j/k*	*ZmNF-YA1; ZmNF-YA4; ZmNF-YA6; ZmNF-YA7; ZmNF-YA11; ZmNF-YA13; ZmNF-YA14;*
*zma-miR169d*	No predicted targets
*zma-miR169e*	No predicted targets
*zma-miR169l*	*ZmNF-YA1; ZmNF-YA4; ZmNF-YA6; ZmNF-YA7; ZmNF-YA11; ZmNF-YA13; ZmNF-YA14;*
*zma-miR169o*	*ZmNF-YA4; ZmNF-YA7; ZmNF-YA11; ZmNF-A14;*
*zma-miR169p*	*ZmNF-YA1; ZmNF-YA4; ZmNF-YA6; ZmNF-YA7; ZmNF-YA11; ZmNF-YA13; ZmNF-YA14;*
*zma-miR169q/n/m*	*ZmNF-YA4; ZmNF-YA7; ZmNF-YA11; ZmNF-A14;*

**Figure 5:**
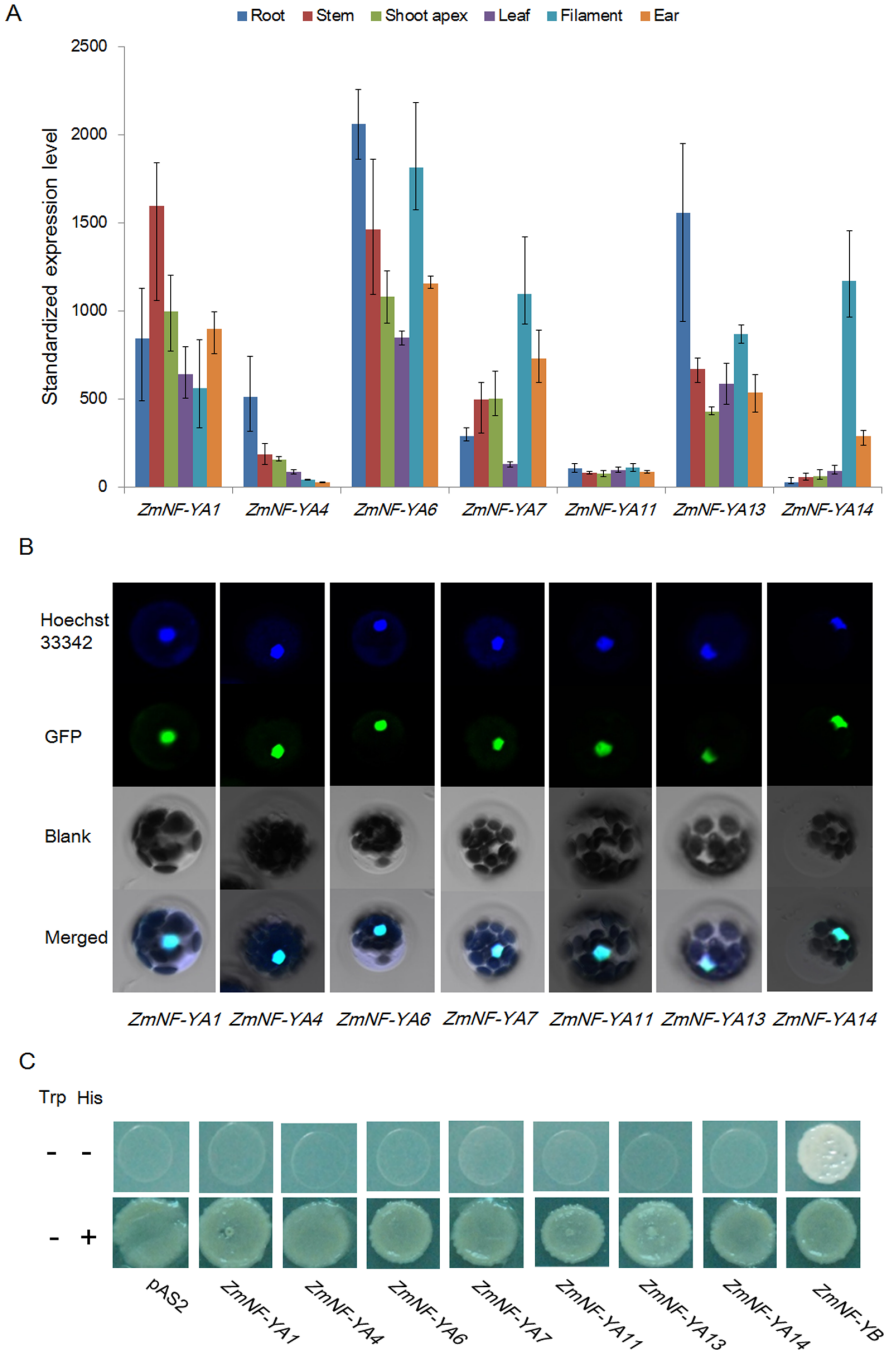
Expression profiles of seven maize *ZmNF-YA* genes and localization and transcriptional activity of ZmNF-YA proteins. (A) *ZmNF-YA* expression patterns in maize tissues. Values represent the means of normalized signal reads. Error bars represent standard errors for three independent normalized signal values. (B) Localization of ZmNF-YA:GFP fusion proteins in maize mesophyll cells. Blue: nucleus stained with Hoechst33342; Green: ZmNF-YA:GFP fluorescence. Cambridge blue: merged images. (C) Transcriptional activation by ZmNF-YAs in a yeast two-hybrid system. Negative control: pAS2 vector. Positive control: ZmNF-YB.

To understand the roles of these *zma-miR169* target genes in maize development, their expression profiles were determined by maize gene chip data in roots, stems, leaves, shoot apices, filaments, and ears. *ZmNF-YA1*, *ZmNF-YA6*, and *ZmNF-YA13* were expressed highly in all seven tissues, whereas *ZmNF-YA11* was expressed at low levels in all seven tissues, *ZmNF-YA4* was expressed at low levels in six tissues (except roots), and *ZmNF-YA14* was expressed at low levels in six tissues (except filaments). *ZmNF-YA7* showed intermediate expression in stems, shoot apices, filaments, and ears (Figure 5A).

NF-YAs act as a DNA sequence-seeking component of the trimeric transcription factor and are expected to be localized to the nucleus where it can combine with the NFYB/C dimer to create the active transcription factor [17]. To verify ZmNF-YA nuclear localization, ZmNF-YAs were fused to green fluorescent protein (GFP) and expressed in maize mesophyll cell protoplasts (Figure 5B). Fluorescence signals of ZmNF-YA:GFP proteins were detected only in the nuclei, indicating that ZmNF-YA proteins are localized in the nucleus. To determine if ZmNF-YAs conferred transcriptional activation, ZmNF-YAs were expressed in the yeast two-hybrid system. Based on the results of the yeast transcriptional activation experiment, ZmNF-YAs lacked transcription activation, but ZmNF-YB can activate reporter gene expression demonstrating that NF-YA need bind to NF-YB/C and form triple complex to function as transcription factors (Figure 5C).

### Responses of *zma-miR169s* and their targeted *ZmNF-YAs* to abiotic stress

To survive in the natural environment, complex gene networks evolved in plants in which miRNAs mediate transient gene silencing to facilitate rapid adaptation to adverse environmental conditions [4], [5]. Several stress-regulated miRNAs have been identified in model plants under various biotic and abiotic stress conditions. In particular, *miR169* is a general abiotic stress-responsive miRNA [10], [14], [18], [25], [26]. Roots bear the brunt of water stress caused by drought or high salinity. Previous studies showed that maize roots are more sensitive than shoots to salt stress [27].

To explore the regulatory mechanism of *zma-miR169/ZmNF-YA* modules in roots under abiotic stress, maize plantlets were subjected to drought and salt stress and treated with ABA. Short-term (0∼48 h) and long-term (15 days) *zma-miR169* and *ZmNF-YA* expression profiles in maize roots were determined. Mature *zma-miR169d* and *zma-miR169e* were not detected, indicating that they might not express in the early developmental stage in maize.

**Figure 6:**
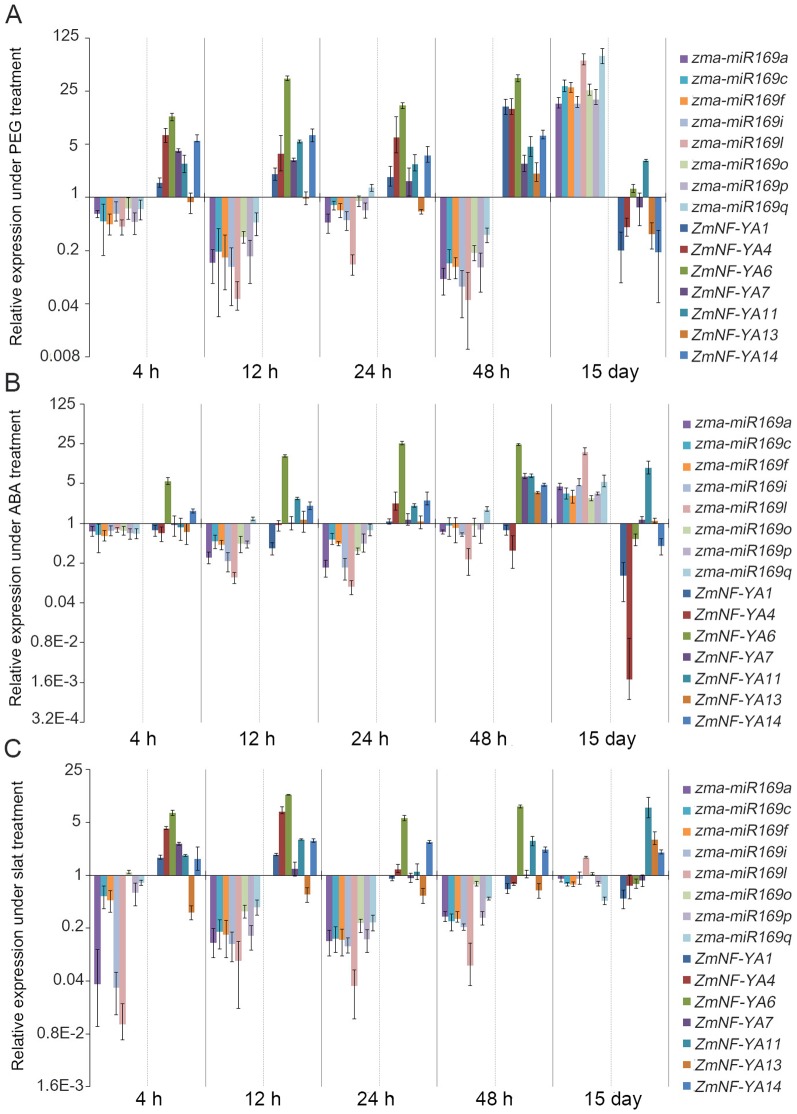
Expression profiles of mature *zma-miR169*s and *ZmNF-YA*s in roots in response to PEG (A), ABA (B), and salt (C). Expression levels of U6 and Tub5 were used as internal references for expression of zma-miR169s and ZmNF-YAs, respectively. Values represent the means and the error bars represent standard errors for three independent experiments.

Drought stress was mimicked by treatment of seedlings with polyethylene glycol (PEG). All *zma-miR169* genes were dramatically downregulated during 0∼48 h with corresponding upregulation of *ZmNF-YA1*, *4*, *6*, *7*, *11*, and *14* expressions. *ZmNF-YA13* was slightly downregulated within 24 h and then upregulated at 48 h. We concluded that *ZmNF-YA13* expression was initially repressed by drought stress. Expression of all *zma-miR169* genes increased dramatically in response to long-term treatment with corresponding downregulation of *ZmNF-YA1*, *4*, *7*, *13*, and *14* expressions. However, *ZmNF-YA11* expression was not downregulated and maintained a similar level of expression over both short-term and long-term treatments. *ZmNF-YA11* appeared to escape regulation by *zma-miR169* over the long term, which may be the result due to expression of *ZmNF-YA11* and *miR169s* in different tissues or cells (Figure 6A).

All *zma-miR169* gene family members were dramatically downregulated in roots by ABA treatment during 0∼48 h and then upregulated in the 15 day. Expression of *ZmNF-YA6* and *ZmNF-YA14* showed a corresponding upregulation in the short term and downregulation in long term, indicating that *ZmNF-YA6* and *ZmNF-YA14* were regulated mainly by zma-miR169s. *ZmNF-YA11* expression was upregulated in both the short term and long term, indicating that *ZmNF-YA11* expression was regulated by ABA in addition to *zma-miR169*. *ZmNF-YA1* and *ZmNF-YA4* were repressed slightly in the short term and dramatically repressed in the long term, indicating that *ZmNF-YA1* and *ZmNF-YA4* were co-regulated by ABA and *zma-miR169s*. *ZmNF-YA7* and *ZmNF-YA13* expression levels showed almost no change in the short term or long term with only upregulation at 48 h (Figure 6B).

All of *zma-miR169* gene family members were dramatically downregulated by salt stress in the short term. Long-term salt stress caused slight downregulation of *zma-miR169a*, *c*, *f*, *i*, *p*, and *q* expression and upregulation of *zma-miR169l*. These results indicated that *zma-miR169* expression was repressed at an early stage but recovered to normal levels over the long term. *ZmNF-YA6* and *ZmNF-YA14* showed a corresponding upregulation in both the short term and long term, indicating that *ZmNF-YA6* and *ZmNF-YA14* were regulated mainly by miRNA169s. *ZmNF-YA13* expression was downregulated in the short term, indicating that *ZmNF-YA13* was regulated mainly by salt over miR169. *ZmNF-YA11* was upregulated in both the short term and long term, similar to the effects of PEG and ABA treatments. Expression of *ZmNF-YA1*, *4*, and *7* was clearly upregulated within 12 h, indicating that *ZmNF-YA1*, *4*, and *7* were regulated mainly by *miR169* at the early stage (Figure 6C).

Taken together, the results show that *zma-miR169* gene family expression was rapidly and dramatically repressed in maize roots in response to abiotic stress, and the repression was gradually released over time, then that expression was upregulated in the long term. The expression of most *ZmNF-YA* transcripts targeted by *zma-miR169* showed an inverse pattern with increased expression in the short term followed by decreased expression over time. These results revealed that *zma-miR169/ZmNF-YA* modules may provide a short-term rapid response to various stress conditions in roots. Over the long term, the responses of different *NF-YA* genes to stress were not the same. Expression of some *NFYA* genes remained under regulation by *zma-miR169/ZmNF-YA*, while others were released from *zma-miR169* regulation. These results indicated that the expression of the *zma-miR169* gene family members and their target genes was dynamically regulated at different time points during stress and that the regulation changed under continuous stress, suggesting the action of an adaptive process in stressed plants.

Notably, *ZmNF-YA14* should be a significant member, which was regulated by *zma-miR169s* in response to drought and salt stress and ABA treatment throughout the 0 h∼15 day time period. *ZmNF-YA6* was the *ZmNF-YA* family member with the greatest increase in expression in the short term in response to drought and salt stress and ABA treatment. We concluded that *ZmNF-YA6* encodes an important rapid response factor for abiotic stress in plants. In contrast, *ZmNF-YA11* was an aberrant member of the family that was upregulated from 0 h through 15 days in response to drought and salt stress and ABA treatment.

### Maize root growth is promoted by drought and ABA but repressed by salt

**Figure 7:**
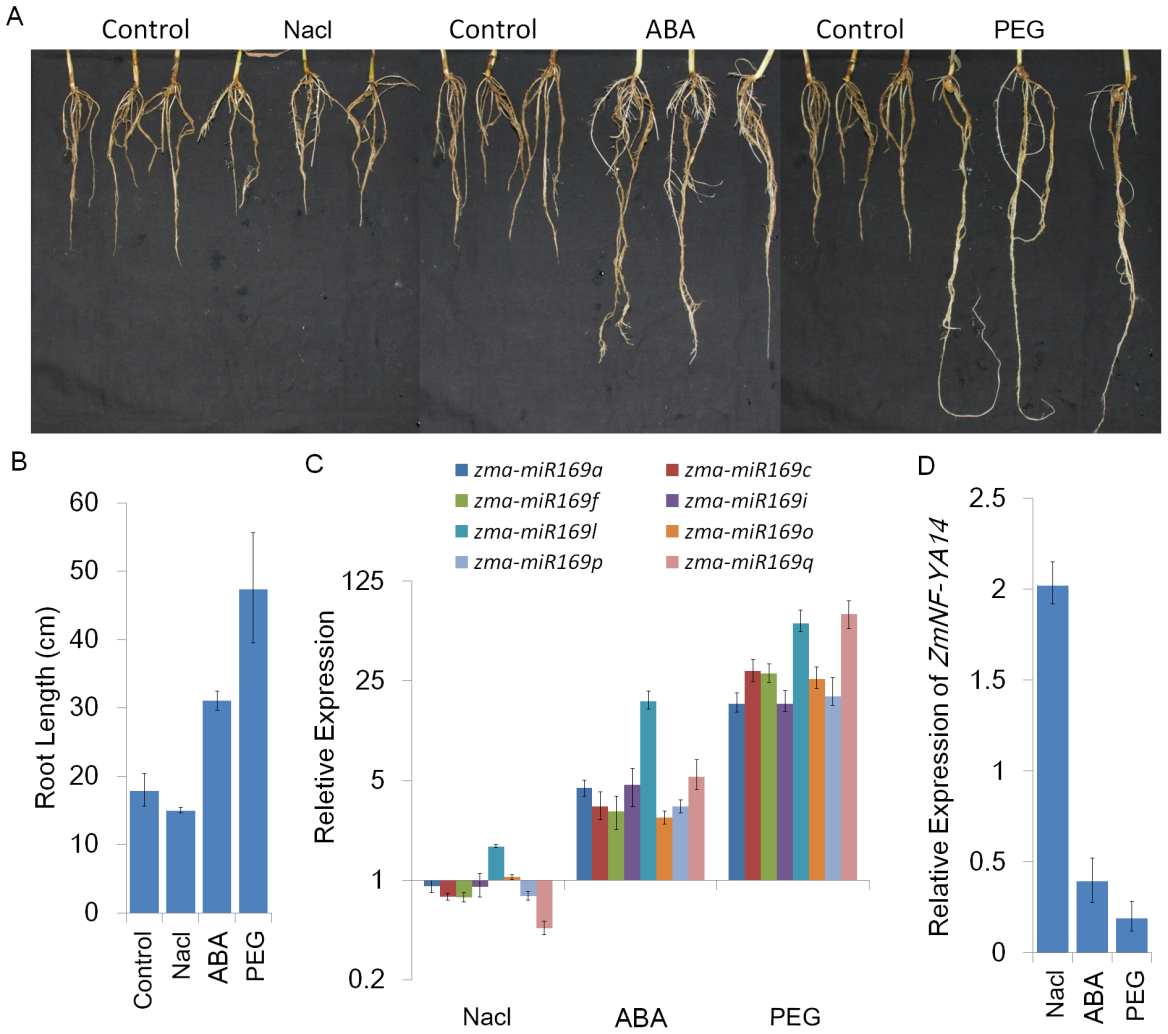
Maize root growth and *zma-miR169/ZmNF-YA14* module expression in response to treatment with NaCl, ABA, or PEG. (A) Root phenotypes in response to treatment with NaCl, ABA, or PEG for 15 days. (B) Root lengths of seedlings treated with NaCl, ABA, or PEG. (C) Relative expression levels of *zma-miR169* genes in seedlings treated with NaCl, ABA, or PEG. (D) Relative expression levels of *ZmNF-YA14* in seedlings treated with NaCl, ABA, or PEG.

Maize seedlings that were treated with PEG and NaCl for 15 days repressed growing and the oldest leaves withered, but no obvious changes were observed for leaves treated with ABA (Figure S5). The effects of the treatments on root length were investigated. The average roots lengths were 19 cm for control seedlings, 15 cm for NaCl-treated seedlings, 31 cm for ABA-treated seedlings, and 47 cm for PEG-treated seedlings. Root lengths were clearly increased by ABA and PEG, but decreased by NaCl (Figure 7A). The roots of the PEG-treated plants were approximately threefold longer than those of the control plants (Figure 7A and 7B). The expression profiles of mature zma-miR169s showed a strong association with root length (Figure 7C). Under NaCl treatment, *zma-miR169* genes were slightly downregulated, with the exception of *zma-miR169l* and *zma-miR169o*, which showed a slight increase in expression. The expression levels of all *zma-miR169* family members were upregulated 3∼17-fold and 17∼70-fold after 15 days of ABA and PEG treatment, respectively (Figure 7C). *ZmNF-YA14* expression was repressed by ABA and PEG treatment and induced by salt stress (Figure 7D). These results suggest that the *zma-miR169/ZmNF-YA14* module might be involved in root growth in response to abiotic stress.

## Discussion

### Expression patterns of *miR169* in different species in response to abiotic stress

Sophisticated mechanisms have evolved in plants to cope with a variety of environmental stress conditions. The expression of many plant genes is controlled by a complex regulatory cascade. MiRNAs can act as switches in such cascades to activate transcription factors, which then activate or repress downstream target genes important for abiotic stress tolerance. Understanding stress response regulatory networks regulated by small RNAs can provide new insights for the genetic improvement of plant stress tolerance. The *miR169* family is the largest and most conserved miRNA family in plants. To date, the miRBase database version 20 (www.mirbase.org) contains 18 maize *miR169* family members. These members contain very similar or identical mature sequences (Figure 1) and predicted target genes (Table 2). Previous reports have shown that *miR169* is upregulated by drought [10], [28], low temperature [9], [11], high soil salinity [14], [29], and UV-B radiation [26]. These results showed that *miR169* family members are involved in plant responses to abiotic stress. Most studies have analyzed *miR169* expression profiles in response to stress in whole plants. The few studies that have separately analyzed *miR169* expression in different tissues such as roots and shoots have shown that *miR169s* respond differently in different tissues during stress. For instance, *miR169s* displayed tissue-specific regulation during N-deficiency stress in maize with upregulation of miR169s in leaves, but downregulation in roots [3]. Differential regulation of miRNAs in different tissues is likely to be important for adaption to stress, and tissue-specific regulation may be overlooked in analyses of whole plants. Roots bear the brunt of water stress caused by drought or high salinity. In this study, we showed that three abiotic stress conditions caused changes in the expression levels of *zma-miR169* and their target genes in maize. The responses of *zma-miR169s* and their targets to drought,salt and ABA treatment were well regulated in roots (Figure 6). The expression patterns of the *ZmNF-YA* mRNAs targeted by *zma-miR169* were inversely related to the levels of *zma-miR169* expression, suggesting that *ZmNF-YA* expression in roots in response to abiotic stress is controlled predominantly by *zma-miR169s* (Figure 6).

All *zma-miR169* family members were rapidly and dramatically repressed in maize roots when exposed in abiotic stress. *ZmNF-YA6* expression showed the strongest increase in expression in the short term in response to drought and salt stress and ABA treatment (Figure 7). *ZmNF-YA6* may be regulated by both *zma-miR169* and stress conditions. We conclude that the *zma-miR169/ZmNF-YA6* module might play a role in rapid responses in roots to various environmental stress conditions. In addition, *ZmNF-YA6* was expressed highly in all tissues (Figure 5A), indicating that it is important for maize development. Further characterization of *ZmNF-YA6* in maize will help us to understand how the *ZmNF-YA6* gene is involved in stress signaling and developmental pathways and how it contributes to abiotic stress tolerance.

### Putative function of the *zma-miR169/ZmNF-YA14* module in long-term abiotic stress responses and root elongation

**Figure 8:**
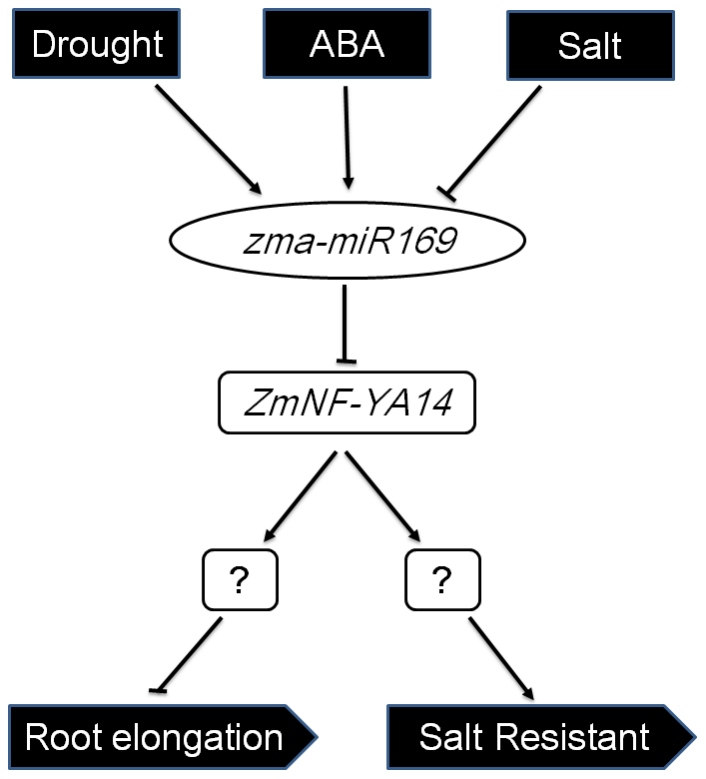
Putative model of zma-miR169/ZmNF-YA14 module response to stress in maize root.

Long-term stress treatments allowed us to examine the stable responses of gene expression level for the stress-adapted plants. To explore the regulatory mechanism of the *zma-miR169/ZmNF-YA* module in maize roots under abiotic stress, the global responses of mature *zma-miR169* members and their targeted *ZmNF-YA* genes to long-term abiotic stress conditions were examined in this study. Expression of *zma-miR169* genes was significantly upregulated by long-term drought and exogenous ABA treatment. In contrast, *zma-miR169* expression remained downregulated after 15 days under salt stress, except for slight upregulation of *zma-miR169l* and *zma-miR169o* (Figure 7C). Root elongation was inhibited in control plants by 15 days of salt stress and promoted by 15 days of drought stress and exogenous ABA treatment (Figure 7A). *ZmNF-YA14* expression decreased in response to drought and exogenous ABA, but increased significantly in response to salt. *ZmNF-YA14* expression exhibited a perfect linear relationship with root elongation, indicating that *miR169/ZmNF-YA* modules are likely to be involved in this regulation. Our results show that plants can respond differentially to drought or salt stress probably via the *miR169/NF-YA* module in roots for adaptation (Figure 8). Plants promote root growth in response to drought to obtain more water, but retard root growth in response to salt to avoid ion absorption and osmotic stress.

## Methods

### Plant culture and sampling

The maize line B73 was used in this study. B73 seeds were sterilized with 10% hydrogen peroxide, washed three times with distilled water, soaked in distilled water for 6 h, and then germinated for 2 days at 28°C in darkness between two layers of filter paper moistened with distilled water. Seedlings approximately 2 cm tall with consistent growth were transferred to coarse silica sand and grown under a temperature cycle of 28°C/24°C during a 14/10 h light/dark circle. Seedlings with two visible uniform leaves were selected to be cultured in water and transferred to half-strength solution and then to full-strength solution (solution compositions are listed in Table S5) the next day. The seedlings with three leaves were used for abiotic stress treatments. The seedlings were subjected to three treatments: 125 mM NaCl for salt stress, 16% PEG to mimic drought stress, and 50 μM ABA to simulate osmotic stress. For short-term experiments, seedlings were sampled at 0 h, 4 h, 12 h, 24 h, and 48 h. For long-term experiments, seedlings were sampled after 15 days. Untreated seedlings served as a control.

### Bioinformatics analysis

We obtained NFYA family and miR169 family sequence data separately from the Plant Transcription Factor Database v2.0 (Center for Bioinformatics, Peking University, China, http://planttfdb_v3.cbi.edu.cn/) and the miRBase (University of Manchester, http://www.mirbase.org/), respectively. Sequence alignments were conducted using DNAMAN software (Lynnon Biosoft, Pointe-Claire, Quebec, Canada), and MEGA5 software was used to generate the NFYA phylogenetic tree analysis and reliability values at each branch representing bootstrap samples (1000 replicates). Prediction of mature miR169 family member targets was performed online with the PsRNA server using relatively strict rules (http://plantgrn.noble.org/psRNATarget/), including a maximum expectation value of 3.5, a length for complementarity scoring (hsp size) of 21 bp, a target accessibility (UPE) of 25, a flanking sequence length around the target site of 17 bp in upstream/13 bp in downstream for target accessibility analysis, and a range of 9–11 nt central mismatch leading to translational inhibition.

### Plasmid construction

Total RNA was isolated from B73 and reverse transcribed to generate cDNA for gene cloning. Pre-miR169 family members and NFYAs with 3′UTRs were cloned into T-vector (Transgene pEASY-Blunt Cloning Vector; Beijing TransGen Biotech, Beijing, China) for sequencing. We used a recombination reaction to construct an overexpression vector. The expression vector was linearized with restriction enzymes, and specific primers were used to clone the genes from the T-vectors. Cloned gene fragments and linearized vectors were purified (D2500; Omega Bio-Tek, Norcross, GA, USA). The gene fragments and linearized vector were combined at a molar ratio of 2∶1, and the recombination reaction was carried out using 15 μl of GBclonart solution (Genebank Bioscience Inc., LOCATION) in a 20-μl final reaction mix. The reaction mix was incubated at 45°C for 30 min and immediately chilled on ice for 5 min to stop the reaction; 1.5 μl of the reaction mix was used to transfect Top10 competent cells (Invitrogen, Carlsbad, CA, USA) for vector validation. pTRL2 vector with a double 35 s promoter and a GFP gene was used for subcellular localization. The pCAMBIA3301 vector (Cambia, LOCATION), which carries a ubiquitin promoter, was used to obtain efficient overexpression of pre-zma-miR169 and ZmNF-YA in maize. Validated plasmid vectors were extracted using a PureYield™ Plasmid Midiprep System (A2492; Promega, Madison, WI, USA) for transient transfection.

### Protoplast isolation and transfection

Maize protoplasts were prepared following Sheen's method (http://molbio.mgh.harvard.edu/sheenweb/main_page.html). The middle sections (6–8 cm) of second maize leaves were cut into 0.5-mm strips without damaging the leaves. The leaf strips were digested in filtered enzyme solution (1.5% Cellulase R10, 0.4% macerozyme R10, 0.4 M mannitol, 20 mM KCl, 20 mM MES, pH 5.7, 10 mM CaCl_2_, and 0.1% bovine serum albumin) for 5∼7 h with shaking at 50 rpm. Protoplast transient expression was conducted using a previously described method [30]. Harvested protoplasts were resuspended in MMg solution (0.4 M mannitol, 15 mM MgCl_2_, 4 mM MES, pH 5.7). GFP-fusion vector and miRNA/target gene cocktails were mixed separately with protoplasts for subcellular localization and 5′RACE. An equal volume of PEG solution (40% PEG, v/v, 0.2 M mannitol, and 0.1 M CaCl_2_) was added and mixed with gentle tapping. The DNA/protoplast/PEG mixture was incubated for 10 min at room temperature to complete the transfection. The protoplasts were incubated in WI (0.5 M mannitol, 20 mM KCl, 4 mM MES, pH 5.7). Incubation times were 4∼6 h for RNA analysis and 6–24 h for GFP assays.

### Subcellular localization

A vector containing NFYA fused to GFP was transiently transfected into protoplasts. After 12-h incubation, the protoplasts were stained with 5 mg/ml Hoechst 33342 dye (CalBiochem, San Diego, CA, USA) for 0.5∼2 h to confirm localization of NFYA-GFP in nuclei, which were stained blue with Hoechst 33342 dye. GFP and Hoechst 33342 fluorescence were detected using a confocal laser scanning microscope (SP5; Leica Microsystems CMS, Mannheim, Germany) equipped with a charge-coupled device camera (CoolSnap; RS Photometrics, Tucson, AZ, USA).

### Yeast transcriptional activation activity analysis

Seven *zma-miR169* target genes were cloned into the pAS2 vector using a recombination reaction for transformation into *Saccharomyces cerevisiae* (AH109) by electroporation (Genepulser; Bio-Rad, Hercules, CA, USA). Cell/DNA mixtures were incubated on ice for 10 min, then electroporated according to the manufacturer's instructions for yeast (*S. cerevisiae*, 2-mm cuvette) using settings of 1.5 kV, 25 μF, and 200 Ω. A 2-ml volume of ice-cold 1 M sorbitol with HEPES (10 ml 1 M Sorbitol; 100 μl 2 M HEPES, pH 8) was then immediately added to the cuvette. The cuvette contents were transferred to a sterile 15-ml tube and incubated at 30°C without shaking for 1∼2 h. First round selection was performed on YPD plates lacking Trp, and second round selection was performed on YPD plates lacking Trp and His.

### Total RNA isolation and quantitative PCR

Total RNA was extracted using TRIzol reagent (Invitrogen). DNase (Promega) was used to remove DNA from the total RNA. The DNA-free total RNA (5 μg) was reverse transcribed for first strand cDNA synthesis using the GoScript™ Reverse Transcription System (Promega A5000). The cDNA was diluted threefold for quantitative PCR (qPCR), which was conducted using an AB7500 Real Time System and GoTaq® qPCR Master Mix (Promega A6001). Tubulin was used as an internal control. Gene primers used for quantitative PCR are listed in Table S4.

### miRNA extraction and stem-loop RT-qPCR

Total small RNA was extracted using miRcute (DP501; Tiangen Biotech, Beijing, China). We performed stem-loop pulsed reverse transcription (RT) using a previously described method [31]. RT reactions used SuperScript III reverse transcriptase (Invitrogen) using the following parameters: 30 min at 16°C, 60 cycles of 30 s at 30°C, 30 s at 42°C, 1 s at 50°C, and 5 min at 85°C. qPCR reactions were performed using an AB7500 real-time system (Applied Biosystems, Foster City, CA, USA) and GoTaq® qPCR Master Mix (Promega A6001) using the following parameters: 2 min at 94°C, 45 cycles of 15 s at 94°C, 1 min at 60°C, followed by the melt curve phase. The specific stem-loop RT-qPCR primers are listed in Table S4.

### RLM-5′RACE

We conducted RLM-5′RACE according to the method of Leonardo Alves [32]. Pre-zma-miR169 overexpression vectors (except for *zma-miR169d/e*) and overexpression vectors for seven potential targets with 3′UTR target sites were pooled and co-transfected into maize protoplasts. Total RNA was extracted for 5′RACE assays performed using a 5′-Full RACE Kit (D315; Takara, Kyoto, Japan). Total RNA was linked with an RNA adaptor and reverse transcribed using Reverse Transcriptase M-MLV with random 9-mers to produce cDNA. Nested-PCR was then performed using outer and inner primers to obtain the 3′-end degradation sequence. Amplification products were cloned into a pEASY-Blunt Cloning Vector (Beijing TransGen Biotech) for sequencing. The primers used for nested-PCR are listed in Table S4.

## Supporting Information

Figure S1 **Pre-miR169 sequence aligment in maize.**
(PDF)

Figure S2 **Potential NF-YA family members in maize.** Phylogenetic analysis of petential NF-YAs in maize, the data from Plant Transcription Factor Database v3.0 Center for Bioinformatics, Peking University, China (http://planttfdb_v3.cbi.edu.cn/).Phylogenetic trees were constructed by neighbor joining with complete deletions as implemented by MEGA5 (Molecular Evolutionary Genetics Analysis software, version 5.0) (Tamura et al., 2011). Reliability values at each branch represent bootstrap samples (1000 replicates).
(PPTX)

Figure S3 **ZmNF-YAs protein sequence aligment.**
(DOCX)

Figure S4 **Special PCR products of RLM-5′RACE.** The amplified product of the 5′ RACE on the ZmNF-YAs transcript by the specific nest-PCR primers (Table S4).
(PPTX)

Figure S5 **Maize seedlings were treated by Nacl, ABA and PEG solution for 15 days.** The maize seedlings in their 3 leaf stage to be used for the abiotic stresses treated (125 mM Nacl, 50 μM ABA, %16 PEG) for 15 day.
(PPTX)

Table S1 **miR169 is a very conserved miRNA.**
(XLSX)

Table S2 **Locus of zma-miR169s.**
(XLSX)

Table S3 **Locus of NF-YA genes involved in evolution relationship analysis between maize and rice.** Gene chromosome positions of NF-YAs involved in maize and rice evolution relationship analysis.
(PPTX)

Table S4 **Primers used in this study.**
(XLSX)

Table S5 **Maize water culture solution (pH 6.0).**
(XLSX)
