# Supplementary material for: Family-Wide Survey of miR169s and NF-YAs and Their Expression Profiles Response to Abiotic Stress in Maize Roots
Source: PLoS One. 2014 Mar 14;9(3):e91369. doi: 10.1371/journal.pone.0091369 (PMC3954700; doi:10.1371/journal.pone.0091369)
Supplement: Figure S1 — Pre-miR169 sequence aligment in maize. (PDF) [file pone.0091369.s001.pdf]

Figure 1S. Pre-miR169 sequence aligment in maize

```
zma-miR169a   ..TAGGCTCGGGGACTATGGTGCAGCCAAGGATGACTTGCCGA.....TCGAT.............GTGACGCCTCT...    57
zma-miR169b   ..TAGGCTCGGGGACTACGGTGCAGCCAAGGATGACTTGCCGA.....TCTATCGTCGATCAACGAGCGACGCCTCTGAT    73
zma-miR169c   ..ATGAGGTAGAGAACGGGATGCAGCCAAGGATGACTTGCCGG.....CTCCTGG.....AACCTGGAGGCGTCTC....    64
zma-miR169d   ..GCAATAGGGGCCACTCAGGCTAGCCAAGGA..GACTGCCTATGAACCT.CTCAATGGTCCACACATTCAGGTCCTTTG    75
zma-miR169e   ..GCAATAGGGGCCACTCAGGCTAGCCAAGGA..GACTGCCTACGAACCAACACAAAGGTCCACA.ATTCTGATCCTTTG    75
zma-miR169f   ..ACCAGAGCTGATTCGTTCAGTAGCCAAGGATGACTTGCCTAGGTATATATGCAT..GGGCTATGGCTACATGCCTGAG    76
zma-miR169g   .CAGAGCTAGCC.TGCCTCTGGTAGCCAAGGATGACTTGCCTACATGGTCTC........GCTAGTTCCGGTTGTTGCAT    70
zma-miR169h   .CAATAAGGGCC.TGCCTCTGATAGCCAAGGATGACTTGCCTATGTCCTTT.........GTTTACAAAGGATCAGAATT    69
zma-miR169i   ..GATGAGAGTGGTAGCTCTGGTAGCCAAGGATGACTTGCCTGTGTGCTGGCCACGCTCCCCTCATGCAAGGACCATCTC    78
zma-miR169j   GCGATAAGAGT..CTGTCCAGATAGCCAAGGATGACTTGCCTGTG.GCTTCTTGGCTTGGCTTGGCTCGGGCAAAACCTT    77
zma-miR169k   .CGATGAGAGCA.CTGCTCTGGTAGCCAAGGATGACTTGCCTGTG.GCCTC.........CATCAGTCGCAGAGGACGCT    68
zma-miR169l   ....................GTAGCCAGGGATGATTTGCCTGTG.GTTCCT.......GTCGTTGCCATGCCAATGCCT    51
zma-miR169m   ....................TTAGCCAAGAATGGCTTGCCTATCTCCATTA........TTTTGTTCATCGC....AGG    47
zma-miR169n   ....................TTAGCCAAGAATGGCTTGCCTATCTCCACTG........TTTGGTTCGTCTT...GGGA    48
zma-miR169o   ....................GTAGCCAAGAATGACTTGCCTATGCACGCCC........TCT.GTTGGCAGTTCCGTCC    50
zma-miR169p   ....................GTAGCCAAGGATGACTTGCCGG.CATTATATTTCTTGGCCCGCCGGCAAGTCATCTGGG    58
zma-miR169q   ....................TTAGCCAAGAATGGCTTGCCTATCTCCATTA........TTTGGTCCATCTTCTTGGGA    51
zma-miR169r   ....................GCAGCCAAGGATGACTTGCCGGGTATTTTGAGCATGCATGTTACAGGGGAGCATATGCA    59
Consensus                          agcca g a     tgcc

zma-miR169a   ....TGATCTCG........TCGTCGTCAGATCGTCGCCGATCATCGGCAAGT.TGTTCTTGGCTACACCGTGGCTCCTG    124
zma-miR169b   GTC.TGATCTCGACATCTA.TCGTCGTCAGACCATCATCATCTATCGGCAAGT.TGTTCTTGGCTACACCGTGGCTCCTG    150
zma-miR169c   ......AGCTTG.........CTGTGCTGTGGCTTAGAACTTAGTCGGCAAGTCTGTCCTTGGCTACACCTAGTTCTCTT    129
zma-miR169d   TAAACAAAGGACATAGGCAAGTCATCCTTGGCTATCAGAGGTAGGCCCTTATT...........................    128
zma-miR169e   TAAACAAAGGACATAGGCAAGTCATCCTTGGCTATCAGAGGCAGGCCCTTATT...........................    128
zma-miR169f   AGCCAGTCTCTTGTGACGCTGAGCATGTATAGTGTAGGCA.TGTCTTCCTTGGCTACTCGGAGCGGCTCTAGTCA.....    150
zma-miR169g   GCA..TGCCACTATGCCAGTC..CTGCTG..GGTTTGTGGGCGGTCTCCTTGGCTAGCCTGAGTGGCTCTTGCCTG....    140
zma-miR169h   GTG..GACCTTTGTGTT..............GGTTCGTAGGCAGTCTCCTTGGCTAGCCTGAGTGGCCCCTATTG.....    128
zma-miR169i   GTGTCGACCGACGAGCGAGCGAGCGATCGATCGATGAGAGGATGACGAAGCTTGGGGTGTACGTTGGTCTCTCACGGGCA    158
zma-miR169j   GTGCACGTTTTATTGCTCGCCTCGTGGCC.TCGATCACAGGCAGTCTCCTTGGCTAGTCCGGGCGGGCCCCTTAT.....    151
zma-miR169k   G......TTCTTCTGCTTGTG....GTTG.TCGATCGCAGGCAGTCTCCTTGGCTAGCCCGAGCGGCTCTCATCCA....    133
zma-miR169l   GTACTGTTCGATCTACGGGCAAATCATCC.CTGCTACCG.........................................    89
zma-miR169m   A.....AGCCCTTCCGTGATG....GATGAAATGTGGATGATGGCATCCATTCTT.GGCTAAGT................    101
zma-miR169n   G.....ACCACCCTGATGATGG..GTGTACTGTGTGGATGATGGCAGGCCTTCTT.GGCTAAGC................    104
zma-miR169o   G.....GCAGCCATGGCGACGG..TTGCACAAGGTGAGTTTTTGCGGCGTGGATGATGCAATGTGGCTGCATCGGCAGGT    123
zma-miR169p   GCT.ACGC........................................................................    65
zma-miR169q   G.....ACCTCCCTCATGGTGT..GGATGGAATGTGGATGGTGGCAGGCCTTCT..GGCTAAGT................    106
zma-miR169r   ATC.TGTTTGGAATTTTGAGCCCATACCGTGCGGTGCCTGTCTTCCGGCAAGT.TGTCCTTGGCTACAT...........    126
Consensus

zma-miR169a   CTCCTG...........................              130
zma-miR169b   CTCCTG...........................              156
zma-miR169c   CCTCT............................              134
zma-miR169d   .................................              128
zma-miR169e   .................................              128
zma-miR169f   .................................              150
zma-miR169g   .................................              140
zma-miR169h   .................................              128
zma-miR169i   GTCTCCTTGGCTAGCCCTGACTCACTCTTACCG              191
zma-miR169j   .................................              151
zma-miR169k   .................................              133
zma-miR169l   .................................               89
zma-miR169m   .................................              101
zma-miR169n   .................................              104
zma-miR169o   CTTCTTGGCTAGCCA..................              138
zma-miR169p   .................................               65
zma-miR169q   .................................              106
zma-miR169r   .................................              126
Consensus
```
