# Supplementary figures and images for: Family-Wide Survey of miR169s and NF-YAs and Their Expression Profiles Response to Abiotic Stress in Maize Roots
Source: PLoS One. 2014 Mar 14;9(3):e91369. doi: 10.1371/journal.pone.0091369 (PMC3954700; doi:10.1371/journal.pone.0091369)

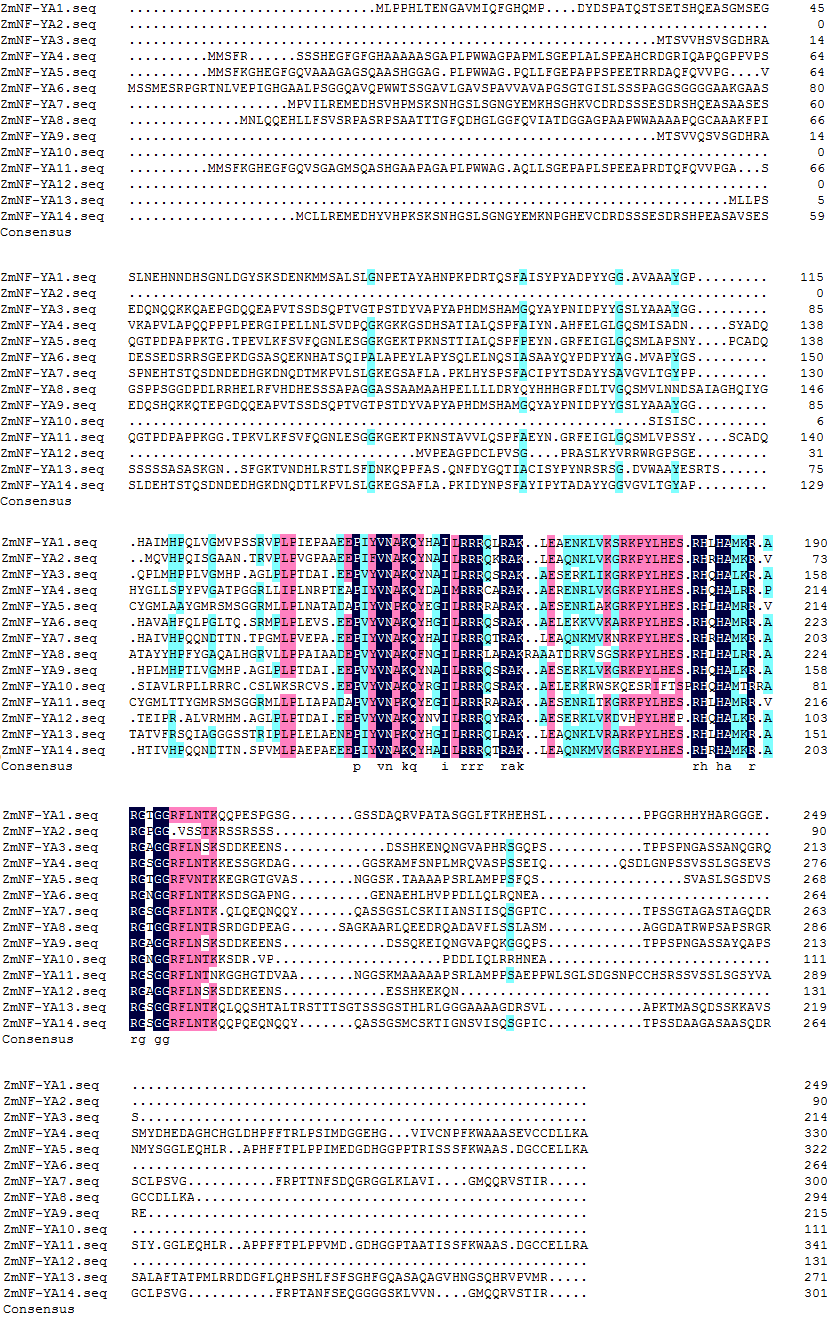


Figure S3. ZmNF-YAs protein sequence aligment

Supplement: Figure S3 — ZmNF-YAs protein sequence aligment. (DOCX) [file pone.0091369.s003.docx]
